# The Efficacy and Safety of the Probiotic Bacterium *Lactobacillus reuteri* DSM 17938 for Infantile Colic: A Meta-Analysis of Randomized Controlled Trials

**DOI:** 10.1371/journal.pone.0141445

**Published:** 2015-10-28

**Authors:** Man Xu, Jiao Wang, Ning Wang, Fei Sun, Lin Wang, Xiao-Hong Liu

**Affiliations:** Department of Pediatrics, the First Affiliated Hospital of Medical School, Xi'an Jiaotong University, Xi'an, Shaanxi Province, People's Republic of China; Scuola Superiore Sant'Anna, ITALY

## Abstract

**Objective:**

To evaluate the efficacy and safety of *Lactobacillus reuteri* DSM 17938 for treating infantile colic.

**Methods:**

A systematic literature retrieval was carried out to obtain randomized controlled trials of *L*. *reuteri* DSM 17938 for infantile colic. Trials were performed before May 2015 and retrieved from the PubMed, EMBASE, Cochrane library, CNKI, WanFang, VIP, and CBM databases. Data extraction and quality evaluation of the trials were performed independently by two investigators. A meta-analysis was performed using STATA version 12.0.

**Results:**

Six randomized controlled trials of 423 infants with colic were included. Of these subjects, 213 were in the *L*. *reuteri* group, and 210 were in the placebo group. *Lactobacillus reuteri* increased colic treatment effectiveness at two weeks (RR = 2.84; 95% CI: 1.24–6.50; *p* = 0.014) and three weeks (relative risk [RR] = 2.33; 95% CI: 1.38–3.93; *P* = 0.002) but not at four weeks (RR = 1.41; 95% CI: 0.52–3.82; *P* = 0.498). *Lactobacillus reuteri* decreased crying time (min/d) at two weeks (weighted mean difference [WMD] = –42.89; 95% CI: –60.50 to –25.29; *P* = 0.000) and three weeks (WMD = –45.83; 95% CI: –59.45 to –32.21; *P* = 0.000). In addition, *L*. *reuteri* did not influence infants’ weight, length or head circumference and was not associated with serious adverse events.

**Conclusions:**

*Lactobacillus reuteri* possibly increased the effectiveness of treatment for infantile colic and decreased crying time at two to three weeks without causing adverse events. However, these protective roles are usurped by gradual physiological improvements. The study is limited by the heterogeneity of the trials and should be considered with caution. Higher quality, multicenter randomized controlled trials with larger samples are needed.

## Introduction

Infantile colic, often described as excessive crying, is prevalent in the first three months of life, in approximately 20% of infants [1]. Because infantile colic usually begins at approximately two weeks of age and improves by month four, previous reports have described it as a benign and self-limiting condition, albeit one that often puts stress on the parents’ mental health [2]. In addition, several observational studies have reported infantile colic to be associated with childhood migraine [3, 4] and attention deficit and hyperactivity disorder [5]. Therefore, an attitude change toward infantile colic is necessary. In fact, even though it is not long lasting, infantile colic should be treated proactively.

The treatment of infantile colic consists of dietary, pharmacological and behavioral interventions and complementary and alternative therapies [2]. Simethicone, a safe, over-the counter drug for decreasing intraluminal gas, has been promoted as an agent to decrease colicky episodes in the 1990s [6], anticholinergic drugs, dicyclomine, herbal teas and cimetropium, have also been used to treat infantile colic, but they did not show obvious improvement in the treated group. Other treatments, such as: placing colicky infants in car-ride simulators or near a clothes dryer or vacuum cleaner [7] also did not reach treatment goal. Besides these treatment protocols, the use of probiotics to treat infantile colic was recently proposed. Several beneficial effects of probiotics on the host intestinal mucosal defenses system have been identified. These include blocking pathogenic bacterial effects by producing bacteriocidal substances and competing with pathogens and toxins for adherence to the intestinal epithelium [8, 9]. Two reviews from 2013 have concluded that the probiotic *Lactobacillus reuteri* (*L*. *reuteri*) DSM 17938 is promising for treating breastfed infants with colic [10, 11]. Although previous results indicated that *L*. *reuteri* was an effective treatment strategy for excessive crying in exclusively breastfed infants with colic, only three small studies found this to be true, and these did not offer sufficient evidence to support probiotics’ use in managing manage colic or preventing excessive infant crying. With the emergence of similar research studies, various results have become available on this topic. In 2014, Sung and colleagues [12] concluded that *L*. *reuteri* was ineffective in both breastfed and formula-fed infants with colic.

Thus, to comprehensively evaluate the efficacy and safety of *L*. *reuter*i (especially DSM 17938) for treating infantile colic, we performed a global search of published randomized controlled trials (RCTs) on this topic. We then applied quantitative analyses with the goal of providing evidence for clinical decision-making.

## Methods

### Inclusion Criteria

We included subjects described in six RCTs performed prior to May 2015. Subjects consisted of infants aged three to six months with term delivery who suffered from colic. All infants were exclusively or predominantly breastfed and formula fed. All the infants enrolled in the studies needed to be consecutively recruited from general pediatrician practices and outpatient populations. The diagnosis of infantile colic accorded with Wessel’s criteria: crying or fussy episodes lasting three or more hours per day and occurring at least three days in the 1 week prior to enrolment. Infantile parents were instructed to maintain and record the daily crying (in minutes) according to the Barr diaries [13] or not. Included infants could not have gastrointestinal disorders, acute or chronic illness, or use of any antibiotic products within seven days prior to the study. There is no limitation on whether infants have allergy to milk protein or a family history of allergy. All analyses in this meta-analysis were based on previous published studies; thus, no ethical approval or patient consent was required.

Infants were assigned at random to receive *L*. *reuteri* DSM 17938 or placebo. The primary end points were treatment effectiveness (defined as percentage of children achieving a ≥ 50% reduction in daily average crying time) and duration of crying (min/d). The secondary end points were infantile growth parameters, maternal mental health (measured with the Edinburgh Postnatal Depression Scale or questionnaire) and adverse events.

### Literature Search

We searched PubMed (1966–2015.5), EMBASE (1974–2015.5) and the Cochrane library (Issue 4 of 12, April 2015) using a searching strategy that combined MeSH/Emtree terms and free text words: Excessive crying, Infantile Colic*, Colic [Mesh/Emtree], Colic, child*, bab*, infant*, kid*, pedia*, pedo*, *Lactobacillus reuteri*, *L*. *reuteri* and *Lactobacillus reuteri* [Mesh/Emtree]. We also searched the following databases in Chinese: CNKI, CBM, WanFang, and VIP. Retrieval dates came from time of database creation to May 2015. To avoid missed terms, we also searched articles using Google Scholar. In addition, we manually checked the references listed at the end of studies to locate potentially eligible research studies.

### Data Extraction and Quality Evaluation

Two investigators independently read the titles, abstracts, and full texts using the following steps: (1) examining titles and abstracts to remove obviously irrelevant studies, (2) retrieving the full texts of potentially relevant trials, (3) examining the full texts for compliance with eligibility criteria, and (4) making final decisions on study inclusion and proceeding to data collection. From the included studies, the investigators extracted baseline information on infant subjects (e.g., treatment strategy, dose, and duration) and detailed methods used in the study design (e.g., publication year, study settings, designs, methods of randomization, allocation concealment, blinding). Disagreements were resolved by discussing them with a third investigator. Some data about crying time was described as median, we translated it into mean to aggregate depending on experience [14], when n≤15, estimated mean $\bar{x}\approx \frac{a+2m+b}{4}$, estimated standard deviation $S^{2}\approx \frac{1}{12}\left({\frac{\left(a-2m+b\right)}{4}}^{2}+{\left(b-a\right)}^{2}\right)$, median (m), low and high end of the range (a and b, respectively); when 15<n≤25, estimated mean $\bar{x}\approx \frac{a+2m+b}{4}$, estimated standard deviation $S\approx \frac{range}{4}$; when 25<n≤70, estimated mean $\bar{x}\approx median$, estimated standard deviation $S\approx \frac{range}{4}$; when 70<n, estimated mean $\bar{x}\approx median$, estimated standard deviation $S\approx \frac{range}{6}$.

Each study was independently assessed for its methodological quality by the previous investigator. The criteria were based on those described in the Cochrane Reviewers’ Handbook 5.1.0. They included selection bias, performance bias, detection bias, attrition bias, and reporting bias domains, random sequence generation, allocation concealment, blinding, incomplete outcome data, selective reporting and other biases.

### Statistical Methods

The meta-analysis was performed using STATA version 12.0 (STATA Corporation, College Station, TX, USA). Relative risk (RR) and weighted mean difference (WMD) were chosen as effective sizes for dichotomous and continuous variables, which were described with a 95% confidence interval (CI). Before analysis, we calculated statistical heterogeneity using I^2^ statistics and assigned low heterogeneity, moderate heterogeneity, and high heterogeneity I^2^ values of 25%–50%, 50%–75% and > 75%, respectively [15]. If the heterogeneity of studies was low (*P* > 0.1, I^2^ < 50%), we adopted a fixed-effects model for quantitative analysis. If the heterogeneity was moderate or high (*P* < 0.1, I^2^ > 50%), possible sources of heterogeneity were analyzed, and subgroup analysis against confounding factors should be divided, after which a random effects model was applied [16]. In addition, investigators conducted sensitivity analyses to explore possible explanations for heterogeneity on the overall pooled estimates. Sensitivity analysis was calculated by omitting a single study in each turn. A statistically significant difference for their conclusion was *P* < 0.05.

## Results

### Process for Selecting Trials

**Fig 1:**
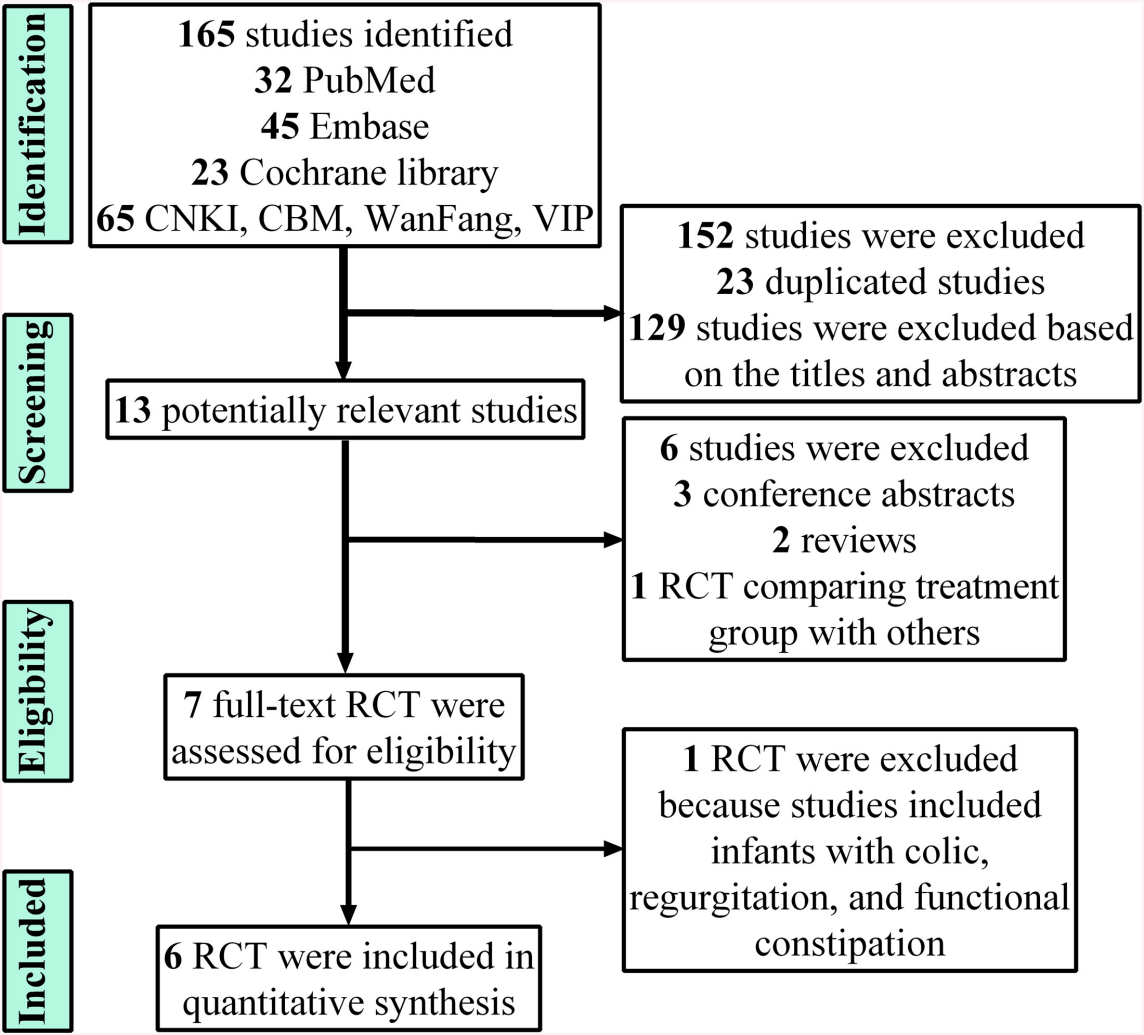
Flowchart of studies included in the meta-analysis.

As shown in Fig 1, 165 potentially relevant studies were identified and screened for retrieval. Twenty-three studies were excluded because of duplications, and 129 studies were excluded after reading their titles and abstracts. Among the remaining 13 studies, three were conference abstracts, two were reviews and one was a RCT comparing *L*. *reuteri* DSM 17938 with simethicone, and therefore excluded. Following this step, seven RCTs were assessed for eligibility. After reading, one RCT that included infantile diseases other than colic was excluded. Finally, six RCTs [12, 17–21] were included in this meta-analysis.

### Characteristics of Included Trials and Quality Evaluation

The main characteristics of the trials included in our meta-analysis are shown in Table 1. The number of infants in RCTs varied from 29 to 167. A total of 423 infants with colic were included in the meta-analysis; of these, 213 were in the *L*. *reuteri* group, and 210 were in the placebo group. All infants were born at term, adequate for gestational age, and aged less than five months at recruitment. Infants in five RCTs were exclusively or predominantly breastfed; only in the Sung study [12] were they breastfed and formula fed.

**Table 1:** Summary of Included Studies.

Study	Patients (n)	Boys/girls	Age (days)	Family history of allergy	Study product	Intervention	Outcomes	Follow-up
Savino 2010 [18]								
*L*. *reuteri* group	25	14/11	28.5±5.25	No reported	Active study product consisted of a suspension of freeze-dried *L*. *reuteri* in a mixture of sunflower oil and medium-chain triglyceride oil supplied in a 5 mL dark bottle fitted with a dropper cap	*L*. *reuteri* at a dose of 10^8^ CFU/day for 21 days	Crying time; number of responders; infants’ intestinal microflora; adverse effects	21 days
Placebo group	25	15/10	32.5±5.25	No reported	The placebo was identical in appearance and taste to the active product but without the live bacteria	Placebo		
Szajewska 2013 [17]								
*L*. *reuteri* group	40	24/16	34.3±12.5	21	*L*. *reuteri* were manufactured and supplied by BioGaia AB (Lund, Sweden) as a fluid in identical bottles and kept refrigerated until use	*L*. *reuteri* at a dose of 10^8^ CFU/day for 21 days	Number of responders; crying time; parental perceptions of colic severity; family quality of life; adverse effects	28 days
Placebo group	40	22/18	38.1±11.7	7	Placebo consisted of an identical formulation in all respects, excluding the live probiotic bacteria	Placebo		
Roos 2013 [19]								
*L*. *reuteri* group	15	9/6	29.8±11.7	No reported	Active study product consists of a suspension of freeze-dried *L*. *reuteri* in a mixture of sunflower oil and a medium-chain triglyceride oil supplied in a 5 ml dark bottle fitted with a dropper cap	*L*. *reuteri* at a dose of 10^8^ CFU/day for 1 month	Number of responders; adverse effects	21 days
Placebo group	14	7/7	29.6±12.9	No reported	Placebo was identical in appearance and taste but without the live bacteria	Placebo		
Sung 2014 [12]								
*L*. *reuteri* group	85	37/48	52.5±20.3	0	*L*. *reuteri* was suspended in oil and dosed once	*L*. *reuteri* at a dose of 10^8^ CFU/day for 1 month	Crying time; number of responders; adverse effects	6 months
Placebo group	82	48/34	48.3±17.5	0	The placebo consisted of maltodextrin in the same oil suspension with the same appearance, color, and taste as the treatment, identically packaged and stored	Placebo		
Mi 2015 [20]								
*L*. *reuteri* group	21	14/7	29.7±13.4	9	An oil-based suspension containing *L*. *reuteri*	*L*. *reuteri* at a dose of 10^8^ CFU/day for 21 days	Number of responders; crying time; parental maternal depression; adverse effects	28 days
Placebo group	21	11/10	28.6±17.6	11	An oil-based suspension containing placebo	Placebo		
Chau 2015 [21]								
*L*. *reuteri* group	27	14/13	42.1±8.9	No reported	The active study product contained *L*. *reuteri* suspended in sunflower oil, medium-chain triglyceride oil, and silicon dioxide	*L*. *reuteri* at a dose of 10^8^ CFU/day for 21 days	Crying time; number of responders; adverse effects	21 days
Placebo group	28	14/14	41.1±9.4	No reported	The placebo contained the same excipient ingredients but without the live bacteria	Placebo		

CFU, colony-forming units; *L*. *reuteri*, *Lactobacillus reuteri* DSM 17 938.

In six RCTs, the oral dose of *L*. *reuteri* was five drops (0.2 × 10^8^ colony-forming units [CFU] per drop) once daily. The duration of treatment differed among studies. Four RCTs [17, 18, 20, 21] treated infants for 21 days, and two RCTs [12, 19] treated them for one month. Active study products in RCTs consisted of a suspension of freeze-dried *L*. *reuteri* DSM 17938 in an oil mixture, whereas the placebos contained the same ingredients but without the live bacteria.

As shown in Table 2, all six RCTs reported random sequence generation from computerized randomization. Allocation concealment was detailed in three studies [12, 17, 21]. Five RCTs [12, 17–19, 21] reported a double-blind design, and one study [20] reported a single-blind design. Intention-to-treat analysis was adopted in three trials [12, 17, 18]. Thus, we determined that these six trials were high quality.

**Table 2:** Quality Evaluation of Included Trials.

Study	Random sequence generation (selection bias)	Allocation concealment (selection bias)	Blinding to participants and personnel (performance bias)	Blinding to outcome assessment (detection bias)	Incomplete outcome data (attrition bias)	Selective reporting (reporting bias)	Other bias
Savino 2010 [18]	Low risk	Unclear risk	Low risk	Low risk	Low risk	Low risk	Low risk
Szajewska 2013 [17]	Low risk	Low risk	Low risk	Low risk	Low risk	Low risk	Low risk
Roos 2013 [19]	Low risk	Unclear risk	Low risk	Low risk	Low risk	High risk	High risk
Sung 2014 [12]	Low risk	Low risk	Low risk	Low risk	High risk	Low risk	Low risk
Mi 2015 [20]	Low risk	Unclear risk	Low risk	High risk	Low risk	Low risk	Low risk
Chau 2015 [21]	Low risk	Low risk	Low risk	Low risk	Low risk	Low risk	Low risk

### Primary End Point

#### Treatment effectiveness

**Fig 2:**
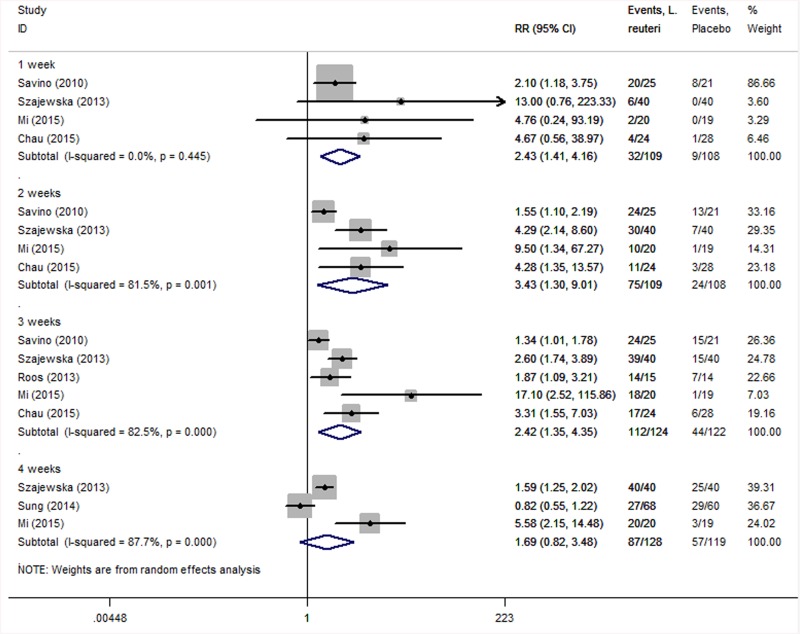
Forest plot comparing treatment effectiveness between *L*. *reuteri* group and placebo group under per-protocol analysis. The statistical method used was the Mantel–Haenszel (M-H) method, the effect measure was RR, and the analysis method was the random effects model.

Five studies [12, 17, 18, 20, 21] compared the treatment effectiveness of *L*. *reuteri* and placebo. As shown in Fig 2, the aggregated results of these studies were divided into four subgroups according to time point. Heterogeneity in one-, two-, three- and four-week subgroups were I^2^ = 0% (*P* = 0.445), I^2^ = 81.5% (*P* = 0.001), I^2^ = 82.5% (*P* = 0.000) and I^2^ = 87.7% (*P* = 0.000), respectively. We adopted a random effects model (using per-protocol analysis), which suggested that *L*. *reuteri* supplementation possibly increased the rate of treatment effectiveness at one week (RR = 2.43; 95% CI: 1.41–4.16; *P* = 0.001), two weeks (RR = 3.43; 95% CI: 1.30–9.01; *P* = 0.012) and three weeks (RR = 2.42; 95% CI: 1.35–4.35; *P* = 0.003) but not at four weeks (RR = 1.69; 95% CI: 0.82–3.48; *P* = 0.158).

**Fig 3:**
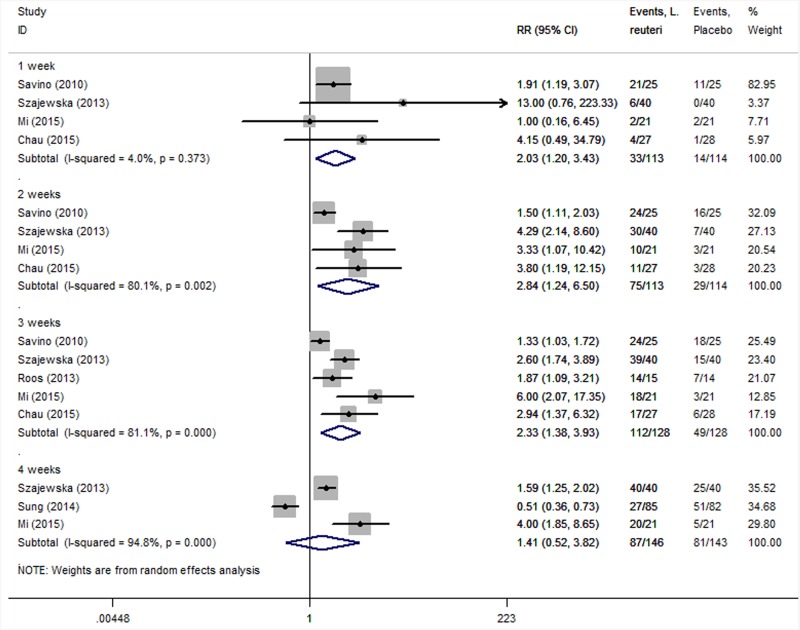
Forest plot comparing treatment effectiveness between *L*. *reuteri* group and placebo group under intention-to-treatment analysis. The statistical method used was the Mantel–Haenszel method (M-H), the effect measure was RR, and the analysis method was the random effects model.

We used intention-to-treatment analysis to aggregate the data. As shown in Fig 3, heterogeneity in the one-, two-, three- and four-week subgroups were I^2^ = 4.0% (*P* = 0.373), I^2^ = 80.1% (*P* = 0.002), I^2^ = 81.1% (*P* = 0.000) and I^2^ = 94.8% (*P* = 0.000), respectively. We adopted a random effects model, and under intention-to-treatment analysis, data showed the same results as in the previous per-protocol analysis: *L*. *reuteri* treatment was able to increase the rate of treatment effectiveness at one week (RR = 2.03; 95% CI: 1.20–3.43; *P* = 0.008), two weeks (RR = 2.84; 95% CI: 1.24–6.50; *P* = 0.014) and three weeks (RR = 2.33; 95% CI: 1.38–3.93; *P* = 0.002) but not at four weeks (RR = 1.41; 95% CI: 0.52–3.82; *P* = 0.498). Meanwhile, the Number Needed to Treat (NNT) is a good epidemiological measure for evaluating the efficacy of *L*. *reuteri*. After calculation (NNT = 1/risk difference [RD]), the NNT of treatment effectiveness was 2.56 (1/0.39, RD was 0.39) at two weeks and 2.23 (1/0.44, RD was 0.44) at three weeks.

**Fig 4:**
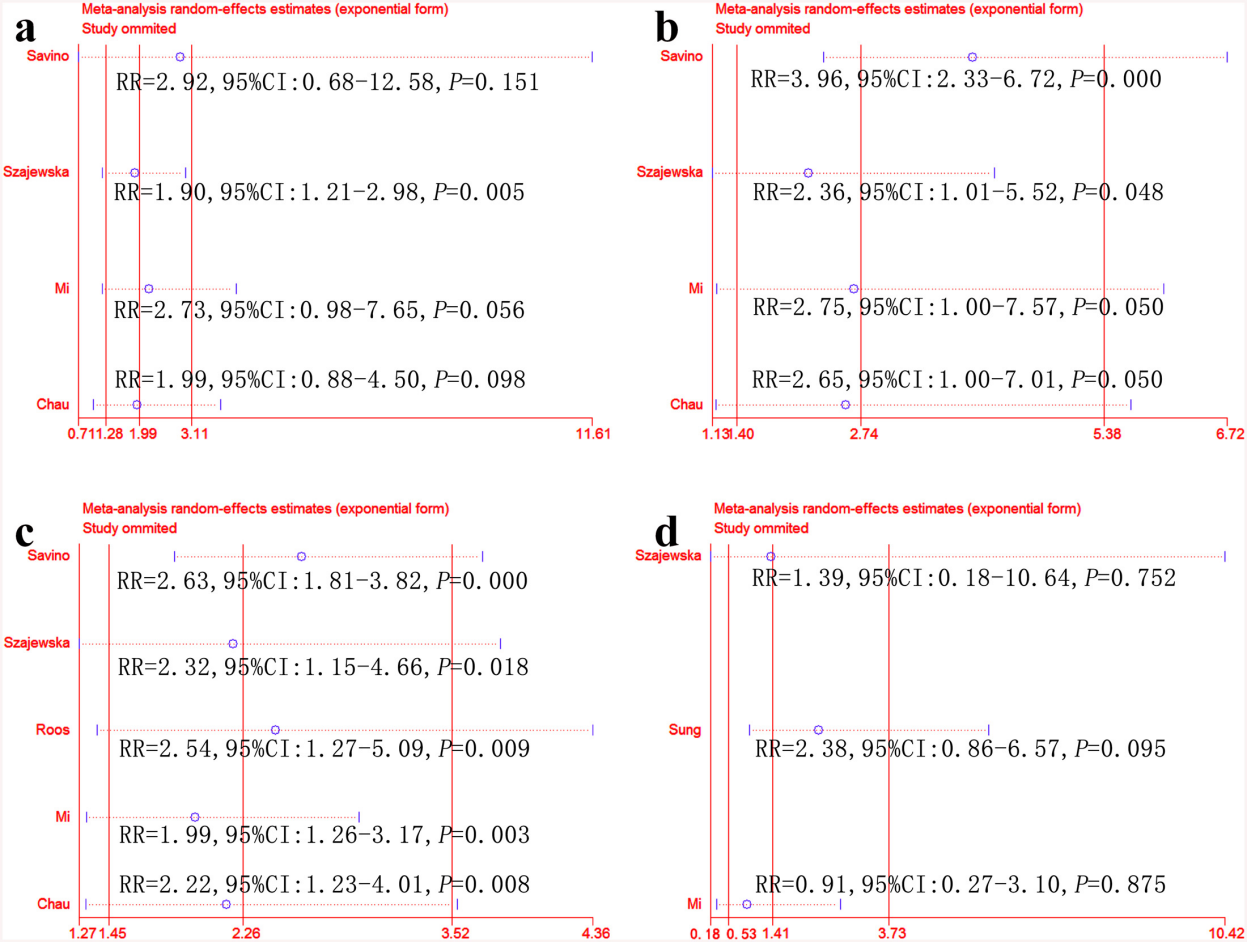
Sensitivity analyses of treatment effectiveness between the *L*. *reuteri* group and the placebo group under intention-to-treatment analysis. One week (a), two weeks (b), three weeks (c) and four weeks (d).

We further performed sensitivity analyses to explore stability. As shown in Fig 4, treatment effectiveness at one, two, three and four weeks changed to varying degrees after removing any one RCT. The studies with the greatest influence on overall results were Savino et al. [18] and Chau et al. [21]. Treatment effectiveness at one week was 2.92 RR (95% CI: 0.68–12.58; *P* = 0.151) after removing Savino et al. [18] and 1.99 RR (95% CI: 0.88–4.50; *P* = 0.098) after removing Chau et al. [21]. These results differed significantly from those of the intention-to-treat analysis.

#### Crying time

**Fig 5:**
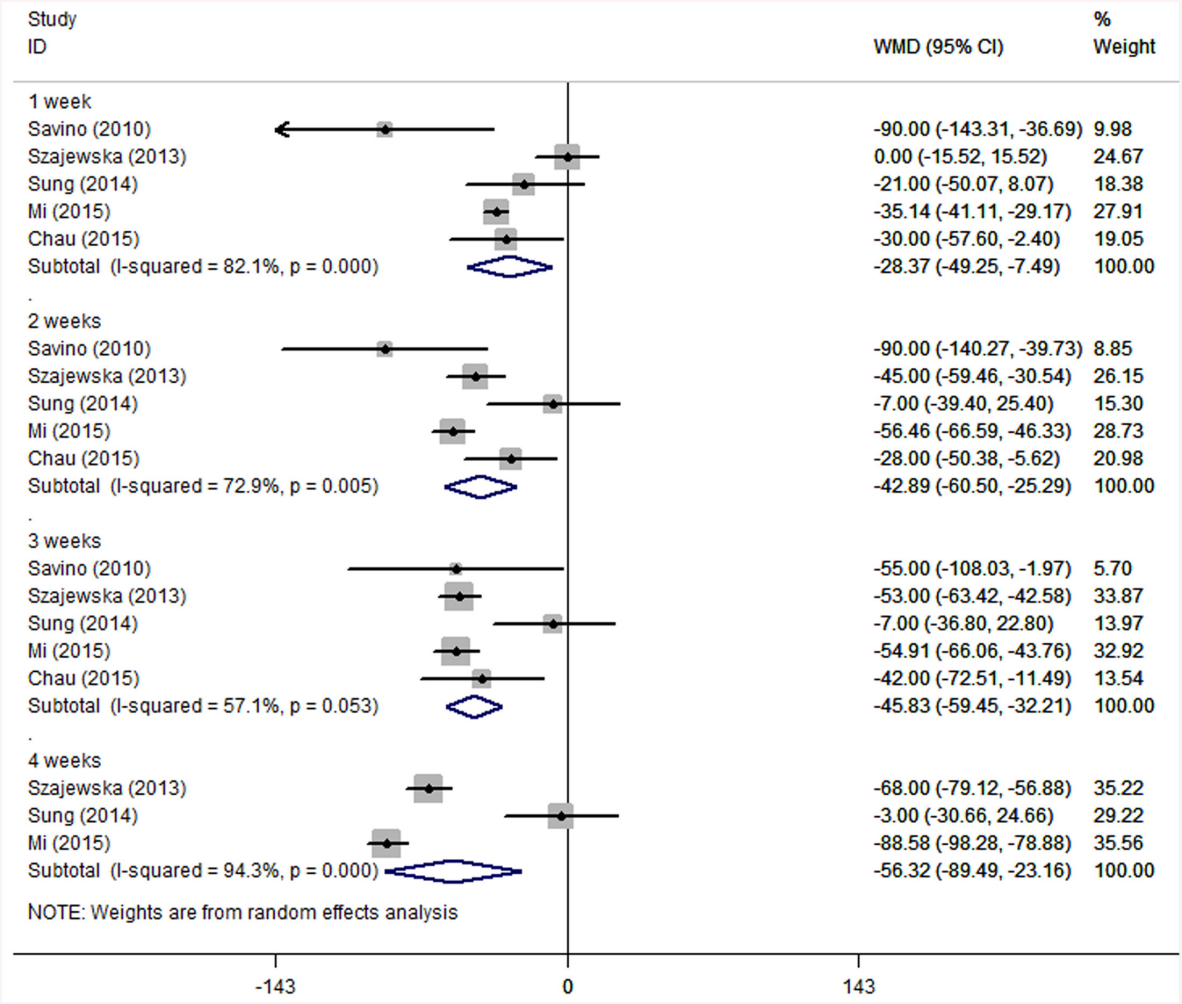
Forest plot comparing crying time between the *L*. *reuteri* group and the placebo group. The statistical method used was Cohen’s d (it is an effect size used to indicate the standardised difference between two means, also widely used in meta-analysis), the effect measure was WMD, and the analysis method was the random effect model.

Five studies [12, 17, 18, 20, 21] reported crying time in the *L*. *reuteri* group and the placebo group. Data in three studies [12, 17, 21] was translated into mean±standard deviation. As shown in Fig 5, the aggregated results of these studies were divided into four subgroups according to treatment time. The heterogeneity in one-, two-, three- and four-week subgroups were I^2^ = 82.1% (*P* = 0.000), I^2^ = 72.9% (*P* = 0.005), I^2^ = 57.1% (*P* = 0.053) and I^2^ = 94.3% (*P* = 0.000), respectively. Thus, we adopted the random effects model for aggregating results. The results demonstrated that *L*. *reuteri* was able to decrease crying time (min/d) at one week (WMD = –28.37; 95% CI: –49.25 to –7.49; *P* = 0.008), two weeks (WMD = –42.89; 95% CI: –60.50 to –25.29; *P* = 0.000), three weeks (WMD = –45.83; 95% CI: –59.45 to –32.21; *P* = 0.000) and four weeks (WMD = –56.32; 95% CI: –89.49 to –23.16; *P* = 0.001).

**Fig 6:**
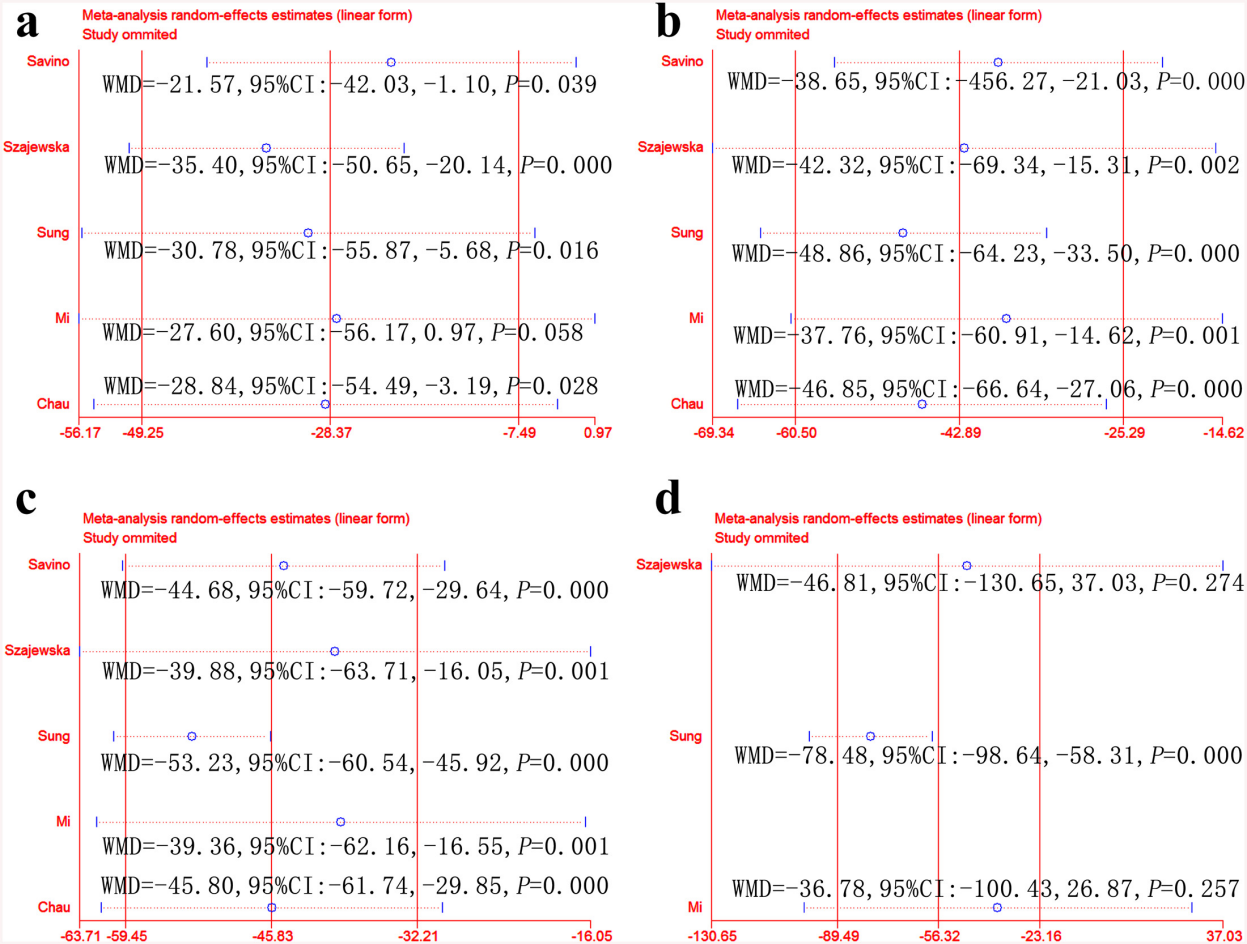
Sensitivity analyses comparing crying time between the *L*. *reuteri* group and the placebo group. One week (a), two weeks (b), three weeks (c) and four weeks (d).

We further performed sensitivity analyses. As shown in Fig 6, crying time at one, two, three and four weeks changed to varying degrees after removing any one RCT. The studies with the greatest influence on the overall results were Szajewska et al. [17] and Mi et al. [20]. Crying time at one week had a WMD of –27.60 (95% CI: –56.17 to 0.97; *P* = 0.058) after removing Mi et al. [20]; crying time at four weeks had a WMD of –46.81 (95% CI: –130.65 to 37.03; *P* = 0.274) after removing Szajewska et al. [17] and a WMD of –36.78 (95% CI: –100.43 to 26.87; *P* = 0.257) after removing Mi et al. [20]. These results differed significantly from those of the intention-to-treat analysis.

Based on the above data, the results showed that *L*. *reuteri* possibly was able to increase the rate of treatment effectiveness for infantile colic at two and three weeks and to decrease crying time at two and three weeks.

### Secondary End Points

**Fig 7:**
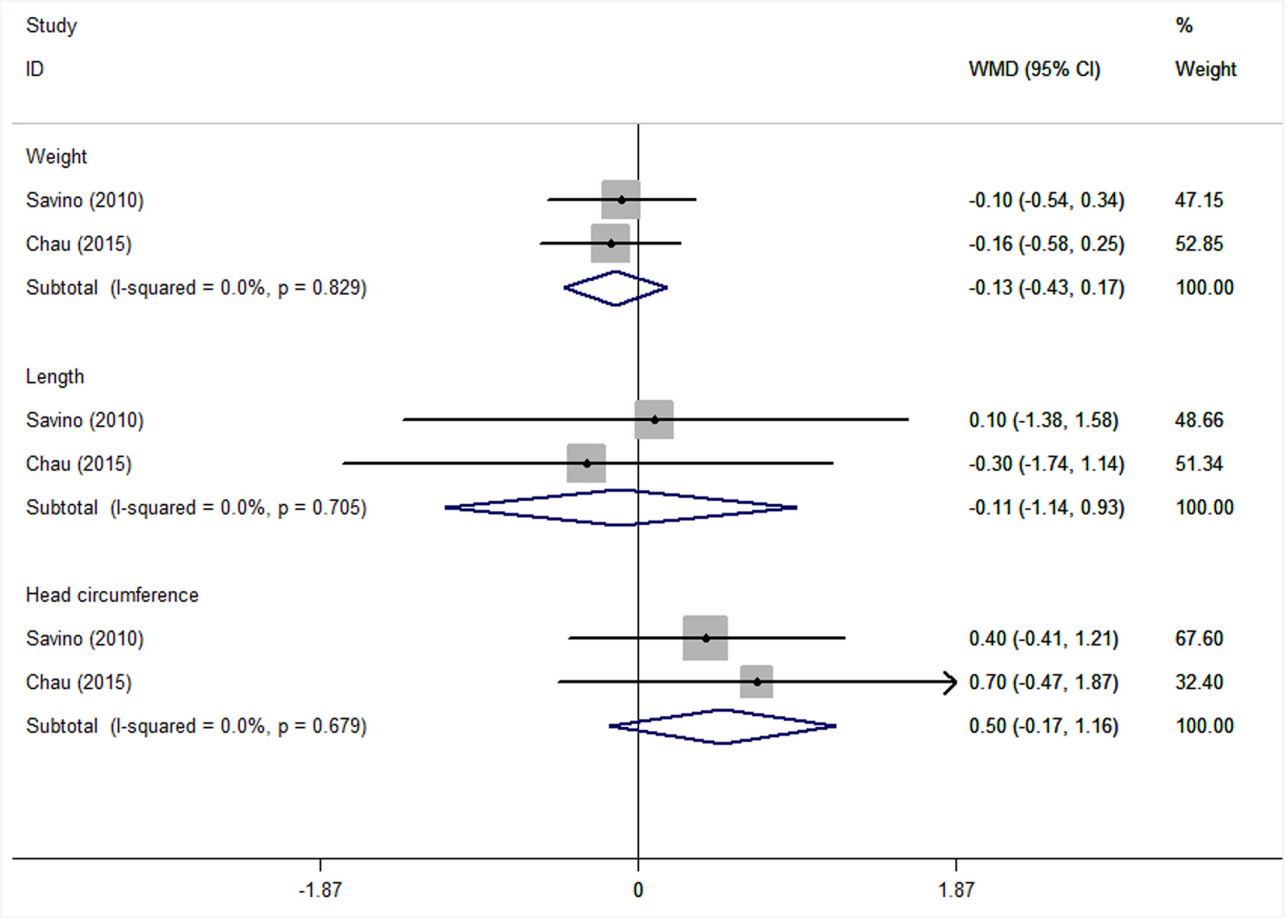
Forest plot comparing growth parameters between the *L*. *reuteri* group and the placebo group on weight, length and head circumference. The statistical method was Cohen’s d, the effect measure was WMD, and the analysis method was the fixed-effects model.

We also compared the growth parameters of both groups based on the two RCTs [18, 21] that reported such data (Fig 7). The results suggested that *L*. *reuteri* supplementation for 21 days to one month did not affect the growth rate of infants: heterogeneities in weight, length and head circumference were I^2^ = 0.0% (*P* = 0.829), I^2^ = 0.0% (*P* = 0.705), and I^2^ = 0.0% (*P* = 0.679), respectively. After we adopted the fixed-effects model, we found that *L*. *reuteri* use did not influence infant weight (WMD = –0.13; 95% CI: –0.43 to 0.17; *P* = 0.385), length (WMD = –0.11; 95% CI: –1.14 to 0.93; *P* = 0.842) or head circumference (WMD = 0.50; 95% CI: –0.17 to 1.16; *P* = 0.143).

It was necessary to assess maternal mental health to reflect the parents’ psychological burden. Only two RCTs [12, 21] reported this information using the Edinburgh Postnatal Depression Scale after the infant received treatment. The results in Mi et al. [20] indicated that *L*. *reuteri* was able to decrease the Edinburgh Postnatal Depression Scale at one to four weeks. Sung et al. [12] reported that this score was similar in the *L*. *reuteri* group and the placebo group at one month.

Adverse events were reported in five RCTs [12, 17, 18, 20, 21]. Savino et al. [18] reported rhinitis in the *L*. *reuteri* group (n = 1) and eczema, fever, otalgy, and gastroesophageal reflux in the placebo group. Four studies [12, 17, 20, 21] reported no adverse events associated with the probiotic therapy or the placebo.

## Discussion

*L*. *reuteri* DSM 17938 is a new probiotic strain that does not carry potentially transferable traits for tetracycline and lincomycin resistance [22]. *L*. *reuteri* is used to treat gastroenteritis in children [23, 24]. The potential mechanism is from the anti-inflammatory effects, because there are number of publications showing anti-inflammatory effects of this probiotic in neonatal animals [25]. Inflammation has been also described in infants with colic [26], and recent evidence suggests that probiotics might offer some benefits to infants with colic as well.

In our meta-analysis, six RCTs contributed to relatively stable results: *L*. *reuteri* DSM 17938 increased the rate of treatment effectiveness at one, two and three weeks, but not at four weeks. *L*. *reuteri* also decreased crying time at one, two, three and four weeks. Sensitivity analyses demonstrated that *L*. *reuteri* increased the rate of treatment effectiveness at two and three weeks and decreased crying time at two and three weeks. Specially, after sensitivity analyses, Savino et al. [18] and Chau et al. [21] in results of treatment effectiveness, Szajewska et al. [17] and Mi et al. [20] in results of crying time at different time points were been found that these studies influenced the stability of results, we drew a comprehensive conclusion based on above analyses, so results from individual study at different time did not affect the final conclusions. Thus, treatment effectiveness and decreasing crying time, two primary outcomes, indicate that *L*. *reuteri* could play a positive role in the treatment of infantile colic, particularly at two to three weeks.

It is important to note that two possible reasons explain why *L*. *reuteri* does not demonstrate a protective role at one and four weeks. First, not only is *L*. *reuteri* not found in every individual, but it is rarely found. Rhoads et al. has studied more than 35 infants with colic and found only 2/34 patients had lactobacilli in the stool before probiotic treatment, and they were both *L*. *gasseri* [26]. Instead, dietary supplementation may be necessary to introduce lactobacilli and to effectively colonize the intestine of healthy infants. After oral intake, colonization of *L*. *reuteri* begins rapidly within days of ingestion before it plays a positive role in the digestive system [27]. Therefore, at one week, the treatment effectiveness and crying times in the *L*. *reuteri* group were equivalent to those in the placebo group. Second, the mean age of the two study groups was 28.5–52.5 days in the *L*. *reuteri* group and 28.6–48.3 days in the placebo group. Therefore, after treatment lasting 21 days or one month, infants were beginning to improve physiologically, and the role of *L*. *reuteri* was not as important to increasing rate of treatment effectiveness and reduction of crying time. This fade-out effect probably explained why there was no difference between the two groups at four weeks. In Sung et al. [12], the mean age of the two groups was greater than that in the other studies: 52.5 days for the probiotic group and 48.3 days for the placebo group, 33.5 days and 35.2 days for the probiotic and placebo group in other five studies. Thus, mean age of the babies was 19 days and 13.1 days greater than other five studies in the probiotic and placebo group respectively. This difference of the studies could explain why Sung et al. [12] was the only one that demonstrated negative results for *L*. *reuteri* treatment. Based on the two points above, we conjecture that *L*. *reuteri* is effective for treating infantile colic if a favorable therapeutic window is chosen.

No serious adverse events were observed in the six studies, and there were no differences in growth parameters between the two groups in our meta-analysis. Even up to the maximum tested dosage of 10^10^ CFU of *L*. *reuteri* per day, no significant differences in standard medical laboratory tests were found [27]. Four studies [12, 17, 20, 21] reported no adverse events associated with the probiotic therapy or the placebo. This indicates that these studies did not prospectively evaluate for the presence of side effects (gastrointestinal, upper respiratory, etc.). Without studies documenting that patients were given a diary card, adverse event frequency cannot be ascertained. This is important because live microorganisms can be linked to side effects such as sepsis and even death in vulnerable infants [28]. Thus, adverse events associated with the probiotic therapy or the placebo should be marked and raised in the next study. Maternal mental health was an indirect indicator of the efficacy differences between the two groups, but only two studies reported this.

Our meta-analysis has several potential limitations that should be taken into account. First, mode of feeding is associated with the occurrence of infantile colic. In this study, infants in five RCTs [17–21] were exclusively or predominantly breastfed; only in Sung et al. [12] were infants breastfed and formula fed. Thus, we could not analyze the effect of feeding mode by subgroup. Second, in most studies, infant crying time recorded by parents varied widely and was based on subjective parental judgments. Third, some data about crying time is described as median, we translate it into mean to aggregate depending on experience. Fourth, three studies [12, 17, 20] reported the information of family history of allergy, but some previous trials indicate that *L*. *reuteri* may have an effect on the immune system and allergic symptoms in children [29], which may have contributed to the differences in the findings. Fifth, Roos et al. [19] focused more on the faecal samples from colicky infants treated with *L*. *reuteri*, rather than the clinical indicator. This study only reported number of responders without documenting crying time, so its result of "improvement" may be more subjective than the other five studies.

In conclusion, the beneficial effects of *L*. *reuteri* DSM 17938 might be demonstrated in infantile colic. The probiotic possibly increases treatment effectiveness and decreases crying time at two to three weeks, without causing adverse events. However, these protective roles are usurped by physiological improvements over time. The conclusions of this study were limited by heterogeneity of the included RCTs and need to be considered with caution. Higher quality, multicenter RCTs with larger patient samples are needed.

## Supporting Information

S1 File PRISMA Checklist. (DOC)
